# Lysophosphatidic Acid-Induced Transcriptional Profile Represents Serous Epithelial Ovarian Carcinoma and Worsened Prognosis

**DOI:** 10.1371/journal.pone.0005583

**Published:** 2009-05-15

**Authors:** Mandi M. Murph, Wenbin Liu, Shuangxing Yu, Yiling Lu, Hassan Hall, Bryan T. Hennessy, John Lahad, Marci Schaner, Åslaug Helland, Gunnar Kristensen, Anne-Lise Børresen-Dale, Gordon B. Mills

**Affiliations:** 1 Department of Systems Biology, University of Texas M. D. Anderson Cancer Center, Houston, Texas, United States of America; 2 Department of Bioinformatics and Computational Biology, University of Texas M. D. Anderson Cancer Center, Houston, Texas, United States of America; 3 Department of Biochemistry, Stanford University School of Medicine, Stanford, California, United States of America; 4 Department of Genetics, Institute for Cancer Research, The Norwegian Radium Hospital, Oslo, Norway; 5 Department of Gynecologic Oncology, The Norwegian Radium Hospital, Oslo, Norway; 6 Institute for Medical Informatics, The Norwegian Radium Hospital, Oslo, Norway; Ordway Research Institute, United States of America

## Abstract

**Background:**

Lysophosphatidic acid (LPA) governs a number of physiologic and pathophysiological processes. Malignant ascites fluid is rich in LPA, and LPA receptors are aberrantly expressed by ovarian cancer cells, implicating LPA in the initiation and progression of ovarian cancer. However, there is an absence of systematic data critically analyzing the transcriptional changes induced by LPA in ovarian cancer.

**Methodology and Principal Findings:**

In this study, gene expression profiling was used to examine LPA-mediated transcription by exogenously adding LPA to human epithelial ovarian cancer cells for 24 h to mimic long-term stimulation in the tumor microenvironment. The resultant transcriptional profile comprised a 39-gene signature that closely correlated to serous epithelial ovarian carcinoma. Hierarchical clustering of ovarian cancer patient specimens demonstrated that the signature is associated with worsened prognosis. Patients with LPA-signature-positive ovarian tumors have reduced disease-specific and progression-free survival times. They have a higher frequency of stage IIIc serous carcinoma and a greater proportion is deceased. Among the 39-gene signature, a group of seven genes associated with cell adhesion recapitulated the results. Out of those seven, claudin-1, an adhesion molecule and phenotypic epithelial marker, is the only independent biomarker of serous epithelial ovarian carcinoma. Knockdown of claudin-1 expression in ovarian cancer cells reduces LPA-mediated cellular adhesion, enhances suspended cells and reduces LPA-mediated migration.

**Conclusions:**

The data suggest that transcriptional events mediated by LPA in the tumor microenvironment influence tumor progression through modulation of cell adhesion molecules like claudin-1 and, for the first time, report an LPA-mediated expression signature in ovarian cancer that predicts a worse prognosis.

## Introduction

The relationship between lysophosphatidic acid (LPA) and the tumor microenvironment remains poorly understood. Although LPA is secreted by ovarian cancer cells [1], found at elevated concentrations in ascites fluid [2]–[5], and LPA receptors are aberrantly expressed in ovarian cancer [6], [7] it is not known whether LPA in the tumor microenvironment activates transcriptional events contributing to the etiology and/or progression of the ovarian cancers. The hypothesis that ascites-borne LPA functions in a pathophysiological context is based on several observations. First, exogenous LPA causes cell proliferation, survival, invasion, migration and production of angiogenic factors likely to contribute to tumorigenesis [8]. Second, LPA receptor expression is altered in ovarian carcinomas [7], [9]. Third, gene expression of LPPs, the naturally occurring enzymes that hydrolyze LPA, is decreased in a significant fraction of ovarian carcinomas [9]. Fourth, enforced expression of LPPs reduces ovarian cancer cell tumorigenesis [10]. Fifth, inhibitors of autotaxin (ATX), the enzyme that generates LPA, reduce both invasion and metastasis in vivo [11].

It is important to determine whether there is a relationship between LPA and patient outcomes since epithelial ovarian cancer (EOC) is the fifth leading cause of cancer mortality for women in the U.S. [12]. Serous epithelial carcinoma accounts for about 70% of EOC and is often associated with peritoneal metastases and poor survival [13]. Serous tumor cells most closely resemble epithelial cells from the fallopian tubes and at least half of all serous tumors are invasive (malignant) with another fraction borderline (low malignant potential) or benign [14]. Peritoneal spread is a primary factor causing ovarian cancer related morbidity and mortality. Since ovarian cancer develops within the peritoneal cavity, its location facilitates a unique version of metastasis as cells shed from the primary ovarian tumor are passively transported through fluid to surrounding tissues, unlike most metastatic tumors [15]. This passive mechanism bypasses many challenging processes facing metastatic tumor cells: intravasion, extravasion, degradation and adhesion.

The latter function, cell adhesion, is critical because strong adhesive anchoring to the extracellular matrix is one of two required signals needed for normal cells to thrive, the other is adequate levels of growth factors [16]. The mitogenic growth factor, LPA, indeed enhances cell growth, viability and proliferation of adherent cells [17]. Furthermore, under stress conditions associated with low fibronectin, Chinese hamster ovary cells stimulated with LPA demonstrated significantly enhanced viability and dramatically reduced apoptosis [18]. This data suggests that LPA may have an additional role to enhance cell adhesion contacts with the extracellular matrix under the stress conditions associated with tumor development and spread.

Normal ovarian surface epithelium rarely expresses the epithelial adhesion molecule E-cadherin, yet increased E-cadherin expression is found in metaplastic ovarian surface epithelium and primary epithelial ovarian carcinomas, suggesting cell adhesion molecules are involved in ovarian neoplastic progression [19]. Cultured ovarian surface epithelial cells frequently resemble fibroblasts, lack cytokeratins and reflect a mesenchymal phenotype conversion. Restored expression of the adhesion molecule E-cadherin reinstates epithelial properties to the cultured cells, marked by tight junctions; [19]. Other adhesive molecules may play a role in the polarity of ovarian epithelial cells , such as claudins, a 24-member family of 20–26 kDa proteins that localize to tight junctions in order to maintain cell-cell adhesion and inhibit any unregulated flow of solutes into tissues.

We sought to better understand how LPA in the microenvironment impacts molecular ovarian cancer progression by examining gene expression changes induced by LPA in ovarian cancer cell lines. Herein we establish a transcriptional profile of 39 LPA-induced genes in OVCAR-3 ascites-derived cells. Applying the available genes from the transcriptional signature to ovarian cancer patient specimens taken during the debulking surgical procedure demonstrated an ability of the signature to predict patient prognosis. This transcriptional profiling uncovered an LPA-mediated increase in claudin-1 (*CLDN1*), an adhesion molecule that is inherently associated with tight junctions, cell adhesion and epithelial cell morphology. The transcriptional increase observed in *CLDN1* correlated with serous ovarian cancer after analysis with ovarian cancer patient profiles, suggesting that LPA may play a molecular role in plasticity of the histology of ovarian cancer. The present study addresses the prognostic value of LPA profiling, provides insight into LPA signaling in the tumor microenvironment and suggests specific LPA-induced genes that may contribute to ovarian cancer progression.

## Methods

### Reagents and materials

LPA (18∶1, 1-oleoyl-2-hydroxy-sn-glycero-3-phosphate) was purchased from Avanti Polar Lipids Inc (Alabaster, AL). OVCAR-3 ovarian epithelial adenocarcinoma and SKOV-3 ovarian serous adenocarcinoma cells were acquired from ATCC (Manassas, VA) and maintained in RPMI (CCSG Media Preparation Facility, The University of Texas M. D. Anderson Cancer Center, Houston, TX) supplemented with 10% FBS (Sigma, St Louis, MO). HT-29 human colon cancer cells were maintained in DMEM (CCSG Media Preparation Facility) supplemented with 10% FBS (Sigma). The claudin-1 antibody was purchased from Zymed (San Francisco, CA) and the GAPDH antibody was purchased from Applied Biosciences/Ambion (Austin, TX).

### LPA-induced gene expression

OVCAR-3 cells were serum-starved prior to stimulation with 20 µM 18∶1 LPA, 20 nM EGF or 2 µM TGFβ for 24 h to mimic long-term LPA influence in the ovarian tumor microenvironment. Pathological LPA concentrations range from 1 to 80 µM and LPA is rapidly degraded in vitro justifying the use of 20 µM LPA in the cultures. RNA was extracted using a Qiagen kit (Qiagen, Valencia, CA) and TRIzol (Invitrogen, Carlsbad, CA) according to the manufacturer's protocol. Gene expression microarray profiling was performed using triplicate sample on Affymetrix GeneChip U133A GeneChips on an HTA system at the Life Sciences Division of the Ernest Orlando Lawrence Berkeley National Laboratory (Berkeley, CA). Data was uploaded to the GeneTraffic Bioinformatics server with GeneTraffic microarray database and analysis software (Stratagene/Agilent Technologies, La Jolla, CA) for raw image analysis, filtering and processing.

### Data analysis for transcriptional signatures

Linear scale data from the GeneTraffic algorithm was transformed to log2. Significance Analysis of Microarrays (SAM) [20 R., and Chu, G. 2001], implemented as the R package *samr* (version 1.20) (http://www-stat.stanford.edu/~tibs/SAM/Rdist/index.html), was used for statistical analysis. The mean expression levels between the LPA-treated and the control groups for significant changes had parameters set such that *resp.type = ‘Two class unpaired’*, *nperm = 100*, *testStatistic = ‘standard’*, and *delta = 0.5*. The final cutoff q-value [21] was 5%. This resulted in 45 up-regulated transcripts, with repeats, giving 39 uniquely-expressed transcripts. Among these genes, 90% had a fold change ≥1.4.

### Categorization of the LPA signature

Gene lists generated from microarray analysis were probed for biological themes using Expression Analysis Systematic Explorer (EASE) analysis tool [22]. This application converts gene lists into biologically relevant categories to aid the interpretation of microarray data. List results were converted into pie graphs using Microsoft Excel to aid visual interpretation.

### Cluster analyses

Hierarchical clustering analyses used Cluster developed by the Eisen lab [23]. Genes were median centered prior to clustering. Cluster processed the data by selecting both genes and arrays and creating average linkage. The display of hierarchical clustering graphs utilized TreeView [23]. For hierarchical clusters, publicly-available ovarian cancer gene expression datasets (GSE6822 [24], GSE6008 [25], GSE10971 and GSE12418 [26]) were downloaded from the NCBI Entrez GEO website (http://www.ncbi.nlm.nih.gov/sites/entrez?db=gds).

### Real-time quantitative PCR

Transcripts in the 39-gene expression profile not previously implicated in LPA signaling were verified with real-time QPCR. mRNA was extracted with GenElute Direct mRNA kit (Sigma Aldrich, St. Louis, MO). Using 300 ng of mRNA as a template, a reverse transcription reaction was performed using Superscript III kit (Invitrogen, Carlsbad, CA) to generate cDNA following the manufacturer's protocol. Quantitative PCR was performed using SYBR Green PCR Master kit (Applied BioSystems, Foster City, CA). Reactions were normalized using the housekeeping gene, β2-microglobulin, and calculations were performed according to previous methods [27]. Primers used were based on algorithm-generated sequences from Primer Bank (http://pga.mgh.harvard.edu/primerbank/) [28]. The results presented used: ATF3 (5′-TCTGCGCTGGAATCAGTCAC-3′ and 5′-GTGGGCCGATGAAGGTTGA-3′); CLDN1 (5′-GCGCGATATTTCTTCTTGCAGG-3′ and 5′-TTCGTACCTGGCATTGACTGG-3′); ETV5 (5′-GCTGTCGTCTTGTAGCCATGA-3′ and 5′-GGGATTCTGATGGGTGGGT-3′); EPAS1 (5′-CGGAGGTGTTCTATGAGCTGG-3′ and 5′-GCTTGTGTGTTCGCAGGAAG-3′); LDLR (5′-TCTGTCTCGAGGGGTAGCTG-3′ and 5′-CAATGTCTCACCAAGCTCTG-3′); β2-microglobulin (5′-GTGGCCTTAGCTGTGCTCG-3′ and 5′-ACCTGAATGCTGGATAGCCTC-3′); MUC1 (5′-AAGCAGCCTCTCGATATAACCT-3′ and 5′-GGTACTCGCTCATAGGATGGT-3′); and SKIL (5′-AATCAATCCAAGACAGATGCACC-3′ and 5′-AGTTTACCACTACTGTGAGCCTT-3′).

### Claudin-1 fluorescent visualization

Fluorescence imaging was done as previously described [17]. In order to asses the localization of claudin-1 after LPA addition, quiescent HT-29 and OVCAR-3 cells were stimulated with LPA (10 µM) for the times indicated and visualized using anti-claudin1 primary antibody (1∶200; Zymed) followed by Oregon Green-conjugated goat anti-mouse immunoglobulin G (1∶500; Invitrogen/Molecular Probes) secondary antibody.

### Boyden chamber migration assay

SKOV-3 cells (2×10^5^) were transfected with reagent only, no siRNA (Mock), RISC-free control siRNA or claudin-1 siRNA. Forty-eight hours later, cells were seeded into the upper wells of a membrane Boyden chamber and migration assay done as described [17]. Results were repeated and confirmed by crystal violet extraction using 10% acetic acid and measuring the OD at 590 nm.

### Two-dimensional cell motility

SKOV-3 cells transfected with siRNA were grown to confluence. Two-dimensional cell motility assay was done as previously described [17]. Cells between monolayers were counted and data is averaged over all experiments. Displayed photomicrograph images (4×) are representative of repeated experiments.

### Western blot assessment of siRNA transfection

Protein from SKOV-3 cells transfected with siRNA was harvested and processed for Western blotting as previously described [17]. Immunoblots were probed with claudin-1 (Zymed) and GAPDH (Applied Biosciences/Ambion) antibodies and visualized with chemiluminescence. Band intensities were normalized to Mock control and represent the ratio of peak values.

### Cell proliferation and viability

SKOV-3 cells transfected with siRNA using in motility studies were examined for viability by detaching cells from 12-well dishes and plating 5, 10 or 15, 20×10^4^ cells in 96-well plates in quadruplicates. Cells were grown for 24 h in 1% medium before adding CellTiter™ Blue reagent (Promega, Madison, WI) to wells to measure viability as previously described [17].

### Cell adhesion

OVCAR-3 cells were transfected with siRNA for 48 h prior to examination for cell adhesion. Cells were seeded at 5×10^4^ cells in 96-well plates in quadruplicates for 2 h before multiple washings with PBS. Calcein-AM solution was then added to each well of the 96-well plate and fluorescence was measured.

### Patient outcome, sensitivity, and specificity analyses

Patient data for disease-specific (DSS) and progression-free survival (PFS) were obtained from clinical reports. For this study, ovarian cancer specimens (N = 79) were obtained at the debulking surgical procedure from patients treated at the Department of Gynecological Oncology at The Norwegian Radium Hospital (NRH) during the period May 1992 to February 2003. Tumor tissues were obtained at primary surgery and were snap frozen in liquid nitrogen. Expression arrays were run on all tumor specimens (N = 79) and clinical data were available for 79 tumors. The median age of the patients at diagnosis was 60 years (range, 38–81). The stage distribution of these ovarian cancers at diagnosis was as follows: stage IIB (1), stage IIC (6), stage IIIB (1), stage IIIC (54) and stage IV (17). The majority of ovarian cancers were of high grade (grade 1 (5), grade 2 (18), grade 3 (56)) and serous in histology (serous (65), endometrioid (4), mucinous (3), undifferentiated (2), mixed (1), clear cell (1), unclassified (3)). Fifty-seven patients developed progressive disease at a median of 15 months (range, 3–126). In those patients without disease progression, follow-up ranged from 77–128 months. Progression-free survival (PFS) was measured from the date of surgery to the date of ovarian cancer progression. Disease-specific survival was measured from the date of surgery to the date of death from ovarian cancer.

### Sensitivity and specificity

The data were plotted in a Kaplan-Meier survival curve format using GraphPad Prism software, which automatically generates p values for the survival curve (GraphPad Software, Inc., San Diego, CA). To ascertain data quality, we employed two supervised classification analyses: linear discrimination analysis (LDA) and K-nearest neighbor classification (KNN). Four of the 60 patients that had reported PFS time had more than 10 missing values, and they were excluded before imputing missing data according to the following protocols: http://www-stat.stanford.edu/hastie/Papers/missing.pdf [29]. Using the statistical parameters, with leave-one-out cross-validation, we achieved high sensitivity (0.88 and 0.94 for LDA and KNN, respectively) and low specificity (0 and 0.25 for LDA and KNN, respectively) for predicting relapse. We also took the 52 patients that have long (>6 mo.) and short (< = 6 mo.) relapse time for the same kinds of supervised classification. Using the same methods, we obtained very low sensitivity (0.29 and 0 for LDA and KNN respectively) and high specificity (0.84 and 0.91 for LDA and KNN, respectively) for predicting short relapse. The overall error rates for LDA and KNN are 0.17 and 0.1, resp., for the first analysis and 0.23 and 0.21, respectively, for the second analysis.

### Statistical analysis

Experimental data was analyzed for statistical differences using an analysis of variance (ANOVA) followed by Bonferroni's Multiple Comparison test or Tukey's test between groups or student's t-test for comparison when the two compared categories have variances assumed equal. Tests applied where indicated and analysis performed using GraphPad Prism software (La Jolla, CA). *p<0.05 **p<0.01 and ***p<0.001 indicate the levels of significance.

## Results

### The 39-gene, LPA-mediated transcriptional signature encompasses carcinogenesis-related transcripts

In order to define a molecular signature regulated by LPA reflective of the ovarian tumor microenvironment, we employed microarray expression profiling using ascites-derived OVCAR-3 ovarian adenocarcinoma cells. Our intention was to uncover LPA-induced transcriptional changes that result from long-term autocrine and paracrine LPA signaling in the tumor microenvironment. For this purpose we stimulated OVCAR-3 cells in culture with LPA (20 µM) for 24 h to mimic chronic exposure. We analyzed the microarray results using the statistical analysis of microarray (SAM) program, which yielded 45 positive hits. The results included several transcript repeats, totaling 39 different genes altogether to comprise a “39-gene” signature (Table 1). LPA has been demonstrated to regulate six of these genes, including *CXCL1* [30], *CYR61* [31], *EGR1* [32], *IL-8* [33], *FN1* [34] and *PLAU* [35], while the remainder were known genes that had unknown associations with LPA. *FN1*, *MUC1*, *SCD* and *THBS1*, appear more than once providing a level of signature validation.

**Table 1:** Gene expression microarray results showing genes comprising the statistically-significant 39-gene LPA transcriptome signature in OVCAR-3.

Probe ID	Symbol	Description	Fold change	q-value (%)
206632_s_at	APOBEC3B	apolipoprotein B mRNA editing enzyme, catalytic polypeptide-like 3B	1.8724	2.377
202672_s_at	ATF3	activating transcription factor 3	2.3955	0
218182_s_at	CLDN1	claudin 1	1.7531	4.5221
204470_at	CXCL1	chemokine (C-X-C motif) ligand 1 (melanoma growth stimulating activity, alpha)	1.7242	4.5221
210764_s_at	CYR61	cysteine-rich, angiogenic inducer, 61	1.722	2.377
204135_at	DOC1	downregulated in ovarian cancer 1	2.2444	0
203498_at	DSCR1L1	down syndrome critical region gene 1-like 1	1.9364	0
201694_s_at	EGR1	early growth response 1	1.446	0
201313_at	ENO2	enolase 2 (gamma, neuronal)	1.5517	0
200878_at	EPAS1	endothelial PAS domain protein 1	1.3329	4.5221
203349_s_at	ETV5	ets variant gene 5 (ets-related molecule)	1.3894	4.5221
205014_at	FGFBP1	fibroblast growth factor binding protein 1	2.5373	0
211719_x_at	FN1	fibronectin 1	2.3993	2.377
212464_s_at	FN1	fibronectin 1	2.2809	2.377
216442_x_at	FN1	fibronectin 1	2.2851	2.377
201631_s_at	IER3	immediate early response 3	1.857	0
202421_at	IGSF3	immunoglobulin superfamily, member 3	1.2985	4.5221
202859_x_at	IL8	interleukin 8	3.9342	0
218963_s_at	KRT23	keratin 23 (histone deacetylase inducible)	1.9927	0
202068_s_at	LDLR	low density lipoprotein receptor (familial hypercholesterolemia)	1.5159	0
220158_at	LGALS14	lectin, galactoside-binding, soluble, 14	1.6646	0
215446_s_at	* LOX	lysyl oxidase	1.4016	2.377
212233_at	MAP1B	microtubule-associated protein 1B	1.9061	0
207847_s_at	MUC1	mucin 1, transmembrane	1.7853	0
211695_x_at	MUC1	mucin 1, transmembrane	2.0686	4.5221
211017_s_at	NF2	neurofibromin 2 (bilateral acoustic neuroma)	1.4298	0
219369_s_at	OTUB2	OUT domain, ubiquitin aldehyde binding 2	1.511	4.5221
211668_s_at	PLAU	plasminogen activator, urokinase	1.8944	2.377
204286_s_at	PMAIP1	phorbol-12-myristate-13-acetate-inducible protein 1	1.4263	0
210138_at	RGS20	regulator of G-protein signaling 20	1.5114	2.377
200831_s_at	SCD	stearoyl-CoA desaturase (delta-9-desaturase)	1.4012	0
200832_s_at	SCD	stearoyl-CoA desaturase (delta-9-desaturase)	1.375	2.377
206884_s_at	SCEL	sciellin	1.823	0
33323_r_at	SFN	stratifin	1.8976	2.377
206675_s_at	SKIL	SKI-like	1.3488	2.377
202856_s_at	SLC16A3	solute carrier family 16 (monocarboxylic acid transporter) member 3	1.6286	2.377
201195_s_at	SLC7A5	solute carrier family 7 (cationic amino acid transporter, y+ system) member 5	1.6561	0
201109_s_at	THBS1	thrombospondin 1	1.9995	0
201110_s_at	THBS1	thrombospondin 1	2.085	0
219058_x_at	TINAGL1	tubulointerstitial nephritis antigen-like 1	1.4088	2.377
217875_s_at	TMEPAI	transmembrane, prostate androgen induced RNA	1.9765	0
201645_at	TNC	tenascin C (hexabrachion)	1.5708	0
202643_s_at	* TNFAIP3	tumor necrosis factor, alpha-induced protein 3	1.8922	0
202644_s_at	* TNFAIP3	tumor necrosis factor, alpha-induced protein 3	1.6207	0
212242_at	TUBA1	tubulin, alpha 1 (testis specific)	1.9468	0

* Denotes decrease in transcript analyzed during verification using real-time QPCR.

To determine the data quality, we created pairwise scatter plots and cross-compared the triplicate microarray data using control (Figure S1A), LPA treated (Figure S1B) and EGF treated (Figure S1C) samples. The scatter plots demonstrate a high degree of reproducibility with microarray replicates, falling close to a 45° reference line. The statistical corroboration enhances the likelihood that transcripts, which have no known connection to LPA are legitimate targets.

For further verification, we performed a time course followed with quantitative RT-PCR on the majority of transcripts with unknown associations to LPA, excluding *CXCL1*, *CYR61*, *EGR1*, *IL-8*, *FN1* and *PLAU*. Thus, we analyzed 88% (N = 29) in OVCAR-3 and 91% in SKOV-3 (N = 30). SKOV-3 cells were selected to determine the generalizability of the results obtained with OVCAR-3 cells. The data shown confirms LPA-mediated modulation of expression among the transcription factors, *ATF3*, *EPAS1*, *ETV5* and *SKIL* (transcriptional co-repressor), appearing in the gene expression microarray data (Figure S1D). During this verification process of the 39-gene signature, we uncovered two genes, *LOX* and *TNFAIP3*, increased in the microarray results but decreased in quantitative RT-PCR (asterix in Table 1 and data not shown). The reason for the differential response in the two assays remains unknown.

Using EASE, we classified the 39 genes comprising the LPA signature into two groups: cellular localization and molecular function. The principal location of one third of 39 genes was the plasma membrane, (Figure S1E). The next most common location was the cytoplasm (26%). Interestingly, over half of the genes in the signature produce secreted proteins (20%) or proteins that are found in regions of the cell that would allow extracellular signaling, like the extracellular matrix (12%) and plasma membrane (30%). When we examined the molecular function of the 39-gene LPA signature, cell-cell communication was the leading represented category (21%) followed by signal transduction (14%) (Figure S1F). A wide variety of molecular functions were also included such as cell growth (13%), adhesion (13%), proliferation (11%), general cell metabolism (9%), lipid metabolism (7%) and products associated with the regulation of either survival or apoptosis (5%).

### The 39-gene, LPA-mediated signature correlates with serous carcinoma and worse prognosis in ovarian cancer patients

Hierarchical clustering using the 39-gene signature and an ovarian cancer training dataset from M.D. Anderson Cancer Center with control/non-malignant and serous EOC revealed a highly similar pattern among patient specimens (data not shown). The training data suggested LPA influences the development of serous EOC and/or the 39-gene signature characterizes serous EOC. To test these hypotheses, we analyzed additional datasets including a test dataset (GSE6008; N = 103) [24] containing normal ovary specimens and ovarian tumors from endometrioid, mucinous, serous and clear cell types.

**Figure 1:**
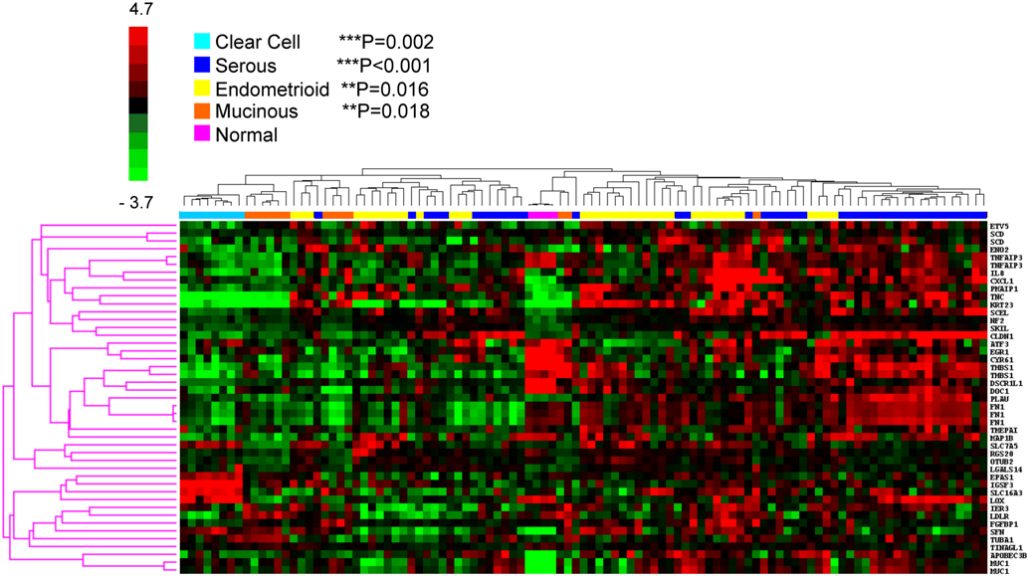
39-gene transcriptional signature clusters serous EOC samples from patient-derived gene expression data. Datasets were downloaded from the NCBI Entrez GEO DataSets website and analyzed for gene expression changes among all included transcripts in the 39-gene signature. Median values of transcripts contained in the LPA gene signature were clustered and average linkages were calculated using the Cluster software. Results were visualized using the TreeView program (see methods). GSE6008, N = 103. The strongly-positive LPA cluster, N = 19, (mostly red) is seen in the bracket on the far right corresponding to serous tumors. Statistical analysis compared normal control samples with ovarian tumor samples of the types indicated and used ANOVA and Tukey's Multiple comparison post test; **P<0.05, **P<0.01 and ***P<0.001.

Hierarchical clustering analysis of GSE6008 demonstrated that the most positive cluster for the 39-gene signature contained only serous specimens (N = 19, 100%) (Figure 1, far right cluster). Within the overall hierarchical clustering results, the gene expression dataset was divided into two major groups (N = 55 and 44) with normal ovary (N = 4) in the middle, separating the two. The group on the right (N = 55) that contained the highly positive LPA-signature cluster and additional clusters varying in LPA-signature-likeness, consisted primarily of serous (N = 29, 71%, P<0.001) or endometrioid (N = 23, 62%, P = 0.016) tumors. The group on the left (separated by normal samples) exhibited expression patterns which deviate from the LPA-signature and consisted of clear cell (100%, P = 0.002), mucinous (77%, P = 0.018), endometrioid (38%, P = 0.016) and a portion of serous (29%, P<0.001) tumors. It is important to note that the majority of the serous tumors on the left side show a distinct expression pattern that deviates from clear cell and mucinous tumors that are negative for the LPA-signature.

To further verify that 39-gene signature characterizes serous EOC, we examined additional ovarian datasets. The dataset GSE6822 (N = 74) [25] contains a majority of serous specimens (N = 46, 62%) and includes benign, borderline and invasive tumors. It is extremely difficult to acquire reliable, high-quality ovarian cancer datasets that include patient outcomes, as few are publicly available. Thus, the dataset we acquired has some incomplete data (gray shaded areas) and missing genes but we nonetheless used it for analysis knowing this limitation. Hierarchical clustering separated a group (N = 19) that was strongly positive for the LPA-signature (Figure S2A, far right cluster). The majority of tumors within this cluster are invasive serous tumors (63%) with a greater proportion of invasive tumors (90%) than other clusters (78%). Among the other clusters, which do not represent a likeness to the LPA-signature, the invasive serous is underrepresented (36% of the remaining tumors), whereas benign serous (83%) and borderline serous (88%) tumors are highly represented. In addition, these other clusters comprise a variety of ovarian tumor types such as clear cell (15%), endometrioid (13%), undetermined origin (11%), mixed (2%) and mucinous (2%), while the LPA-signature is strongly dominated by serous tumors (74%) and has no endometrioid, mucinous or tumors of undetermined origin. Over half of all serous tumor cells are invasive (malignant) and the remaining are borderline (low malignant potential) or benign [14].

Another dataset GSE10971 (N = 37) contains samples from non-malignant fallopian tube epithelium and high-grade serous carcinoma. Hierarchical clustering divided the samples into two groups (data not shown). Among the LPA-positive cluster, all samples were high-grade serous (N = 12, 100%) and among the dissimilar cluster, nearly all samples were non-malignant (N =  24, 96%) (Figure S2B). Only one sample from the latter group was high-grade serous (N = 1, 4%). In summary, the data from our training set combined with the data presented using GSE6822, GSE10971 and GSE6008 datasets suggests that the LPA-signature characterizes serous EOC.

**Figure 2:**
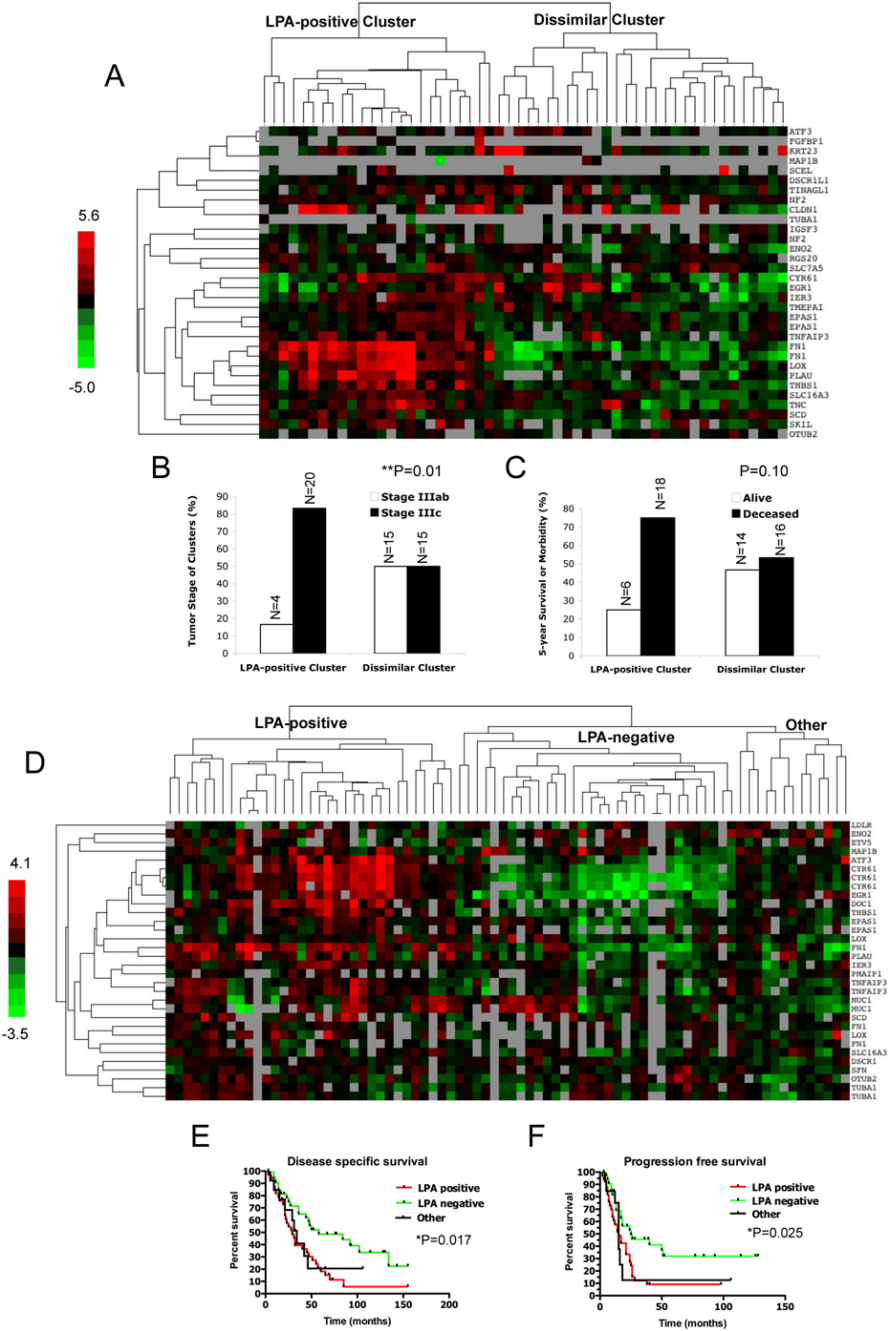
The LPA signature corresponds to worsened outcome in ovarian cancer patients. (A) The patient dataset, GSE12418, N = 54, was downloaded from the NCBI Entrez GEO DataSets website and analyzed for gene expression changes among all included transcripts in the 39-gene signature. Hierarchical clustering using the available genes divided patient samples into two groups, LPA-positive (N = 24) and dissimilar (N = 30) clusters, based largely upon the drivers *THBS1*, *EGR1*, *EPAS1*, *CYR61*, *FN1* and *PLAU*. (B) Separating the clusters based on stage IIIab or IIIc demonstrated a greater portion of samples from stage IIIc patients (N = 20, 83%, P = 0.01) among the LPA-positive cluster whereas the dissimilar cluster is evenly split between stage IIIab and IIIc. (C) Patient samples in the LPA-positive cluster also contained a greater percentage of deceased patients (N = 18; 67%) than the dissimilar cluster (N = 16, 53%). Results from C were not statistically significant. (D) Specimens (N = 79) isolated from patients treated at the Norwegian Radium Hospital (see METHODS) were analyzed by gene expression profiling. Hierarchical clustering of available genes included in the 39-gene signature separated the patients into three groups (LPA-positive, N = 33; LPA-negative, N = 32; Other, N = 13) based largely upon drivers *ATF3*, *CYR61*, *EGR1*, *FN1* and *PLAU*. Kaplan-Meier analysis of the different clusters indicates that the LPA-signature positive cluster has shorter median values for both disease-specific (29 months, P = 0.017) (E) and progression-free (15 months, P = 0.025) survival (F) compared to the LPA-negative cluster (median values of 58 and 25 months, respectively).

Since evidence suggests the 39-gene signature characterizes serous EOC from ovarian datasets (Figure 1, Figure S2A and S2B, and data not shown), we questioned whether it also classified prognosis in ovarian cancers. For this analysis, we acquired two ovarian cancer datasets containing patient outcomes. The first dataset (GSE12418; N = 54) [26] was used to examined the predictive value of the 39-gene signature and it contains serous samples from different stages. Hierarchical clustering separated the specimens into two groups (Figure 2A) based largely upon drivers *THBS1*, *EGR1*, *EPAS1*, *CYR61*, *FN1* and *PLAU*. The LPA-signature-positive cluster (N = 24) contained a greater portion of samples from stage IIIc patients (N = 20, 83%, P = 0.01) than the dissimilar cluster that was evenly split among stage IIIa, IIIb and IIIc (Figure 2B). Although not statistically significant, samples in the LPA-positive cluster also contained more deceased patients (N = 18; 67%) than the dissimilar cluster (N = 16, 53%) (Figure 2C).

The second ovarian cancer dataset containing patient outcomes that we used for the prognosis analysis is a large EOC dataset (N = 79, see METHODS for more details) of specimens acquired from patients treated at the Norwegian Radium Hospital. Briefly, the ovarian cancer specimens were obtained at the debulking surgical procedure from primary tumors. Hierarchical clustering separated the patients into three groups (Figure 2D) based largely upon drivers *ATF3*, *CYR61*, *EGR1*, *FN1* and *PLAU* and to some extent *MUC1*. Clustering the three groups distinguishes the most positive cluster (LPA-positive, N = 33), a negative cluster (LPA-negative, N = 33) and a smaller cluster with differentially expressed genes (Other, N = 13) that has a distinct pattern from the other groups. The Other cluster falls between the LPA-positive and -negative clusters and could represent either a transitional state of development between the two or a distinct set of ovarian tumors driven LPA in the context of other factors.

The LPA-signature-positive cluster comprises significantly shorter median values for both disease-specific (29 months, *P = 0.017) (Figure 2E) and progression-free (15 months, *P = 0.025) survival (Figure 2F) compared to the LPA-negative cluster, which had median values of 58 and 25 months, respectively. Statistical analysis of our findings indicated that this classification had high sensitivity (0.88 and 0.94 for LDA and KNN, respectively) for predicting relapse and high specificity (0.84 and 0.91 for LDA and KNN, respectively) for predicting short relapse. Taken together, our analysis of the 39-gene signature in prognosis of ovarian cancer suggests the signature represents markedly reduced progression-free and disease-specific survival and overall a worsened prognosis.

### Claudin-1 is a biomarker for serous ovarian cancer and transcriptionally regulated by LPA to facilitate ovarian cancer cell adhesion

As therapeutics against LPA begin to enter clinical trials [36], a biomarker capable of distinguishing among subtypes of EOC could provide an extremely valuable classifier to select those ovarian cancer patients who are most likely to benefit from LPA-targeted drugs. Within the 39-gene LPA signature, we sought to determine whether such a biomarker existed. Among hierarchical clustering analyses, particular gene expression transcripts repeatedly drive clustering and patient categorization. More specifically, a combination of *ATF3*, *CLDN1*, *CXCL1*, *CYR61*, *EGR1*, *FN1*, *IL-8*, *MUC1*, *PLAU*, *THBS1* and *TNC* determine the LPA-positive clusters (Figure 1, Figure S2, Figure 2A and 2D, and data not shown) when these genes are included on the microarray chip. Among these drivers, we sought to determine whether a singular transcript is responsible for LPA-positive signature characterization.

**Figure 3:**
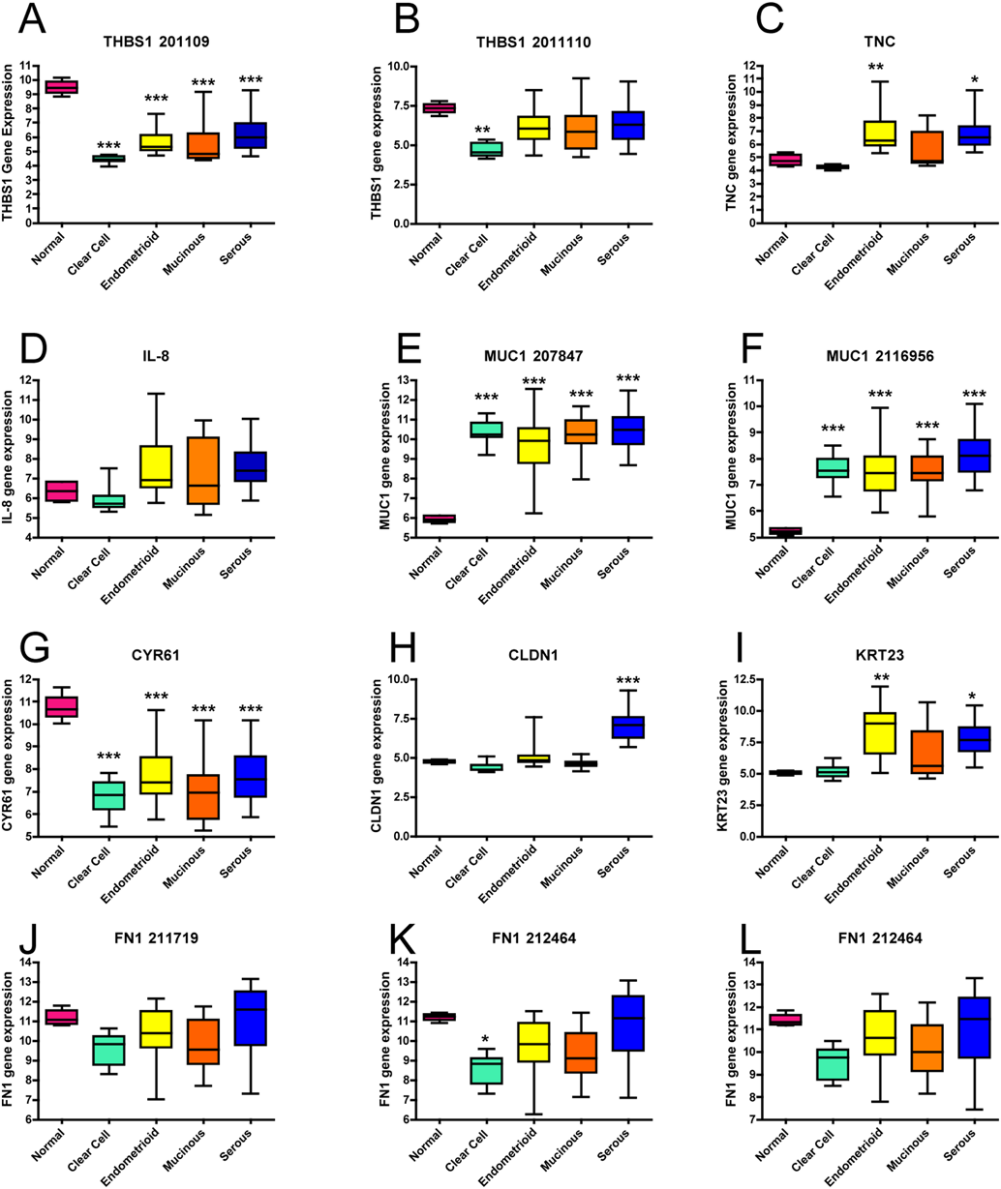
*CLDN1* is a biomarker for serous epithelial ovarian carcinoma specimens. Box-plot analyses of data from GSE6008, N = 103 show the drivers *THBS1* (A, B), *TNC* (C), *IL-8* (D), *MUC1* (E, F), *CYR61* (G), *CLDN1* (H), *KRT23* (I) and *FN1* (J, K, L) normalized comparison of gene expression levels. Among all previously-identified drivers in hierarchical clustering, the data demonstrates that *CLDN1* is the only one which distinguishes serous when compared with normal, clear cell, endometrioid and mucinous (***P<0.001). The box represents the range of values and the center vertical line inside the box represent the median with whiskers representing the extended range. Results were analyzed by ANOVA and Tukey's Multiple comparison post test; **P<0.05, **P<0.01 and ***P<0.001 of ovarian tumor type vs. normal samples.

Using the most complete dataset we acquired, (GSE6008; N = 103) [24], we analyzed the drivers and determined whether classification of LPA-positive serous tumors could be achieved using only *CLDN1*, *CYR61*, *FN1*, *IL-8*, *MUC1*, *THBS1* and *TNC* (Figure S3A). The comprehensiveness of the dataset we selected was based on the large number of samples, numbers of gene transcripts on the microarray and diversity of tissue samples. Using the available cell adhesion proteins, we revisited the data on specimens acquired from patients at the Norwegian Radium Hospital and found that hierarchical clustering of these genes was able to recapitulate the prognosis trends we previously observed for disease-specific and progression-free patient survival (Figure S3B and S3C). Out of the seven drivers in this dataset, claudin-1 (*CLDN1*) was sufficient to differentiate serous from other EOC types (Figure S3D). In comparison, serous carcinoma samples expressed high levels of *CLDN1* whereas mucinous and normal were low and clear cell levels were the lowest. The endometrioid samples included a few patients with high claudin-1 expression (N = 4, 11%) but the majority of samples were at the median level or below (N = 32, 89%). Further analyses showing individual normalized comparison of gene expression levels (Figure 3) demonstrates that *CLDN1* distinguishes serous from normal, clear cell, endometrioid and mucinous and is expressed at elevated levels in serous (P<0.001) (Figure 3H).

**Figure 4:**
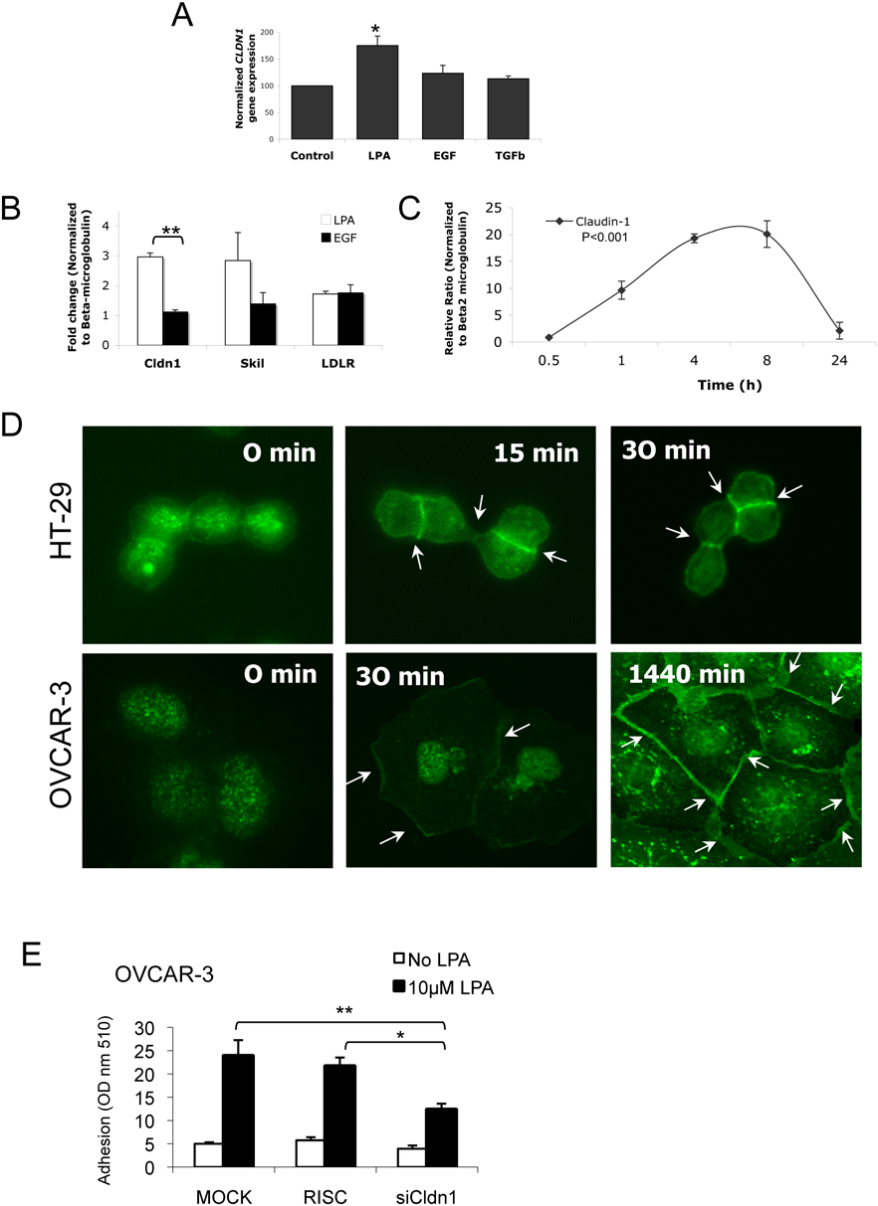
LPA mediates cell adhesion through claudin-1 in OVCAR-3 cells. (A) OVCAR-3 cells were stimulated with either LPA (20 µM), EGF (20 µM) or TGFβ (2 µM) for 24 h before processing for Affymetrix gene expression microarray. *CLDN1* expression was normalized to untreated controls and compared to agonist-stimulated conditions. Results are the average of triplicate samples. Results were analyzed by ANOVA and Bonferroni's post test; *p<0.05, LPA vs. Control. (B) OVCAR-3 cells were stimulated with either LPA (10 µM) or EGF (10 µM) for 24 h and processed for quantitative RT-PCR (see methods) to measure transcriptional activity comparing selected transcripts from the 39-gene signature, claudin-1 (Cldn1), SKI-like oncogene (Skil) and low density lipoprotein receptor (LDLR). Results were normalized to β2-microglobulin and reflect the average of triplicate samples repeated in four independent assays. Results were analyzed by ANOVA and Bonferroni's post test; **p<0.01, LPA vs. EGF. (C) A timecourse of LPA (10 µM) stimulated OVCAR-3 cells was performed for the times indicated using quantitative RT-PCR. Results show a representative experiment that was repeated in OVCAR-3 and SKOV-3 cells. ***P<0.001 for 1,4,8 and 24 h time points compared with normalized control. (D) Quiescent HT-29 and OVCAR-3 cells were stimulated with LPA (10 µM) for the times indicated and visualized using anti-claudin1 primary antibody followed by Alexa goat anti-mouse fluorescent secondary. (E) OVCAR-3 cells were transfected with siRNA targeting claudin-1 for 48 h. Cells were then trypsinized and replated for 2 h in the presence or absence of LPA (10 µM) and analyzed for adhesion. Results were analyzed by ANOVA and Bonferroni's post test; *p<0.05 (RISC with LPA vs. siCldn1 with LPA) and **p<0.01 (Mock with LPA vs. siCldn1 with LPA).

Thus, we next sought to determine the association between LPA and claudin-1 in ovarian cancer cells. Gene expression profiling of *CLDN1* in OVCAR-3 cells after 24 h of stimulation with LPA (20 µM), EGF (20 µM) or TGFβ (2 µM) demonstrated LPA selectively increased *CLDN1* expression (Figure 4A). To confirm these results, we measured mRNA changes using quantitative, real-time PCR and noted that LPA (10 µM) significantly increased *CLDN1* (P<0.01) compared to EGF (10 µM) after 24 h of agonist treatment (Figure 4B). The LPA-mediated increase in *CLDN1* is maximal between 4 to 8 h after LPA (10 µM) treatment, when the relative ratio is approximately 20 times above normalized control (Figure 4C) CLND1 levels remain elevated at 24 h at approximately 1.5 to 3 times control levels (Figure 4C).

We next stimulated OVCAR-3 and HT-29 colon cancer cells with 10 µM LPA for various times and examined effects on claudin-1 localization. HT-29 cells were used as a model for comparison to OVCAR-3 because claudin-1 is a regulator of metastasis in colon cancer [37] and not all cell types express claudin-1. Within 30 min after LPA stimulation, claudin-1 redistributed from the nuclear region to cell junctions in HT-29 cells. In contrast at 30 min of incubation with OVCAR-3 cells, most of the claudin-1 staining was nuclear and only some occurred on the plasma membrane and cell junctions (Figure 4D). Similar claudin-1 staining was seen in OVCAR-3 at 240 min post LPA stimulation (data not shown). However, intense cell junction staining occurred at 1440 min after LPA addition (Figure 4D, bottom right). Taken together, the data suggests that LPA induces increase in mRNA levels for *CLDN1*, which is not replicated by EGF, and LPA localizes claudin-1 to cell junctions in OVCAR-3 and HT-29 cells.

The previous results lead us to question whether the increase and relocalization of claudin-1 altered cell adhesion in OVCAR-3 cells. We thus analyzed cell adhesion in the presence of LPA after manipulating claudin-1 expression. Indeed, 10 µM LPA significantly enhances OVCAR-3 cell adhesion under all transfection conditions (Mock, RISC-free and siCldn1) when compared to transfected cells without LPA present (Figure 4E, white vs. black bars). A reduction in claudin-1 expression significantly reduced the ability of LPA to enhance adhesion (Figure 4E). Taken together, the data suggests that LPA enhances the adhesion of OVCAR-3 cells in part through increased expression and membrane localization of claudin-1.

**Figure 5:**
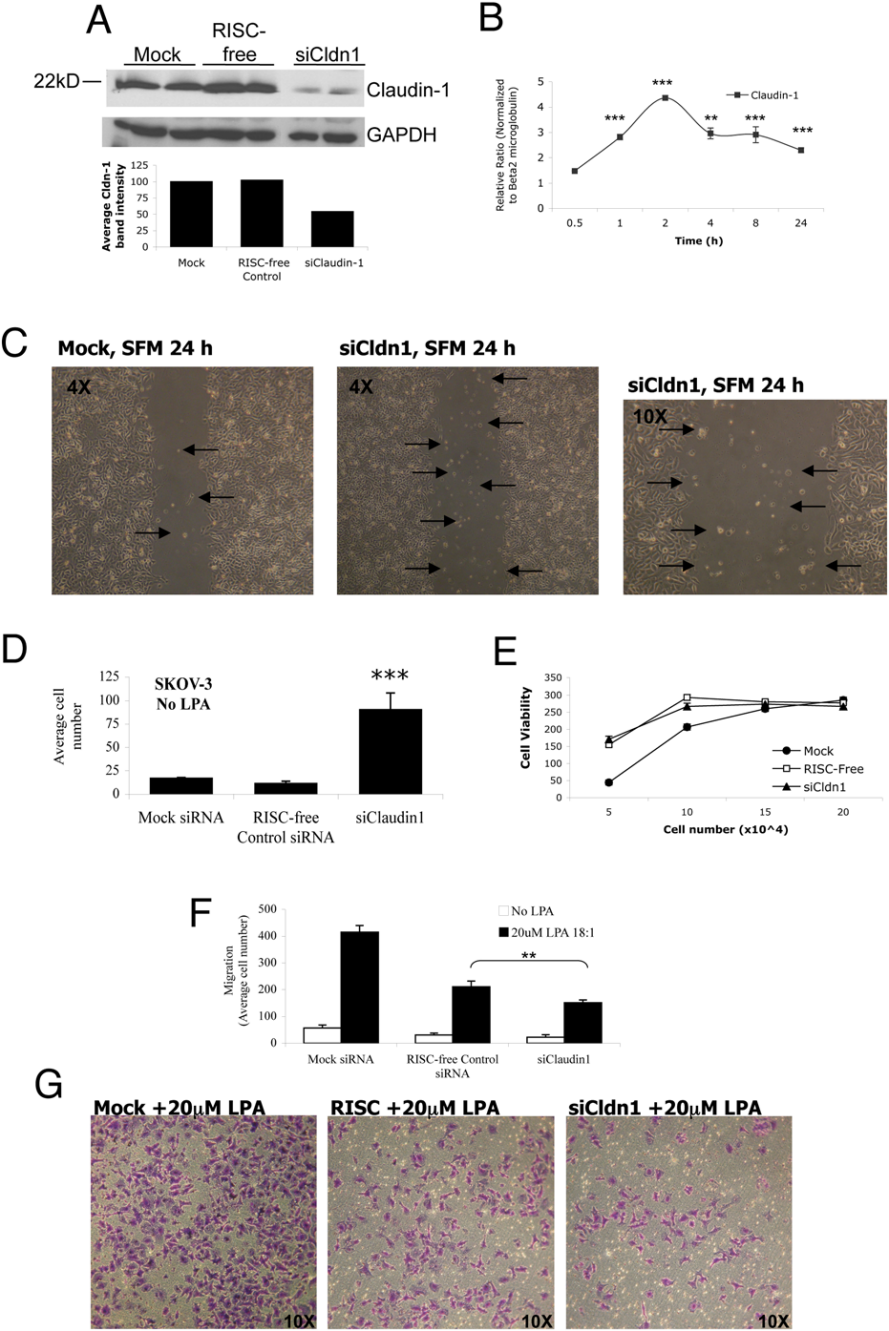
Reduced claudin-1 expression enhances suspended cells and impairs LPA-mediated Boyden chamber migration using SKOV-3 ovarian cancer cells. (A) Protein from SKOV-3 cells transfected with siRNA was harvested and processed for Western blotting. Immunoblots were probed with claudin-1 and GAPDH antibodies and visualized with chemiluminescence. The bands were quantified using ImageJ and normalized to Mock control. Claudin-1 expression was consistently decreased >50%. (B) Quantitative RT-PCR showing modulation of claudin-1 mRNA transcript in SKOV-3 cells in response to LPA treatment for the times indicated. Quantification is normalized to β2-microglobulin, **P<0.01 for 4 h and ***P<0.001 for 1, 2, 8 and 24 h time points compared with normalized control. (C) siRNA transfected SKOV-3 cells were plated in triplicate 12-wells and grown to confluence. Confluent monolayers were then scratched with a pipette tip, rinsed five times in serum-free media (SFM) to remove floating cells, placed in SFM and incubated for 24 h. Microscopic images (4× and 10×) shown are representative (Mock and RISC-free control were identical) of repeated results. Photomicrographs show few cells in the wound (Mock, 4×) versus numerous cells in the wound (siCldn1, 4× and 10×) between monolayers. (D) Quantification of cells in the wounded area from C. ***p<0.001 from ANOVA and Bonferroni's test. (E) Cells from D were examined for viability by detachment from 12-well dishes and plating 5–20×10^4^ in 96-well plates in quadruplicates. Cells were grown for 24 h in 1% medium before adding CellTiter™ Blue reagent to wells overnight to measure colorimetric change indicating cell viability. (F) Approximately 2×10^5^ SKOV-3 cells were plated in 6-well dishes overnight prior to transfection with reagent only and no siRNA (Mock), RISC-free control siRNA or claudin-1 siRNA. Forty-eight hours later, cells were then seeded in Boyden chambers and stimulated overnight with LPA (20 µM) or not (No LPA) to facilitate directed migration through the chamber. (G) Transwells were stained with crystal violet and fields were examined to determine the number of cells that migrated through the pores. Results represent the average of quadruplicate transwells, six fields measured in each transwell and repeated experiments. **p<0.01; LPA-stimulated RISC-free control vs. LPA-stimulated siClaudin-1.

Claudin-1 localizes to tight junctions and plays a role in cell adhesion. However a major reported role of LPA in ovarian cancer cell signaling is induction of motility. To evaluate the role of claudin-1 in ovarian cancer cell migration, we performed a series of functional migration assays after manipulating claudin-1 expression in SKOV-3 cells using siRNA. Claudin-1 levels were decreased approximately 60% (Figure 5A). SKOV-3 cells were selected instead of OVCAR-3 cells because OVCAR-3 cells are not motile and SKOV-3 cells are highly motile and responsive to LPA [38]. SKOV-3 cells are a relevant model system since they also show a similar LPA-mediated increase in *CLDN1* to OVCAR-3 cells at 24 hours (Figure 5B) and they independently verified the 39-gene signature (Table 1 and Figure S1).

Wounding confluent monolayers of SKOV-3 cells stimulated with LPA (10 µM) and transfected with Mock-, RISC-free- or claudin-1 siRNA showed an similar effects on healing in response to LPA after 24 h (data not shown). Similar results were achieved with proliferation assays (data not shown) where no significant changes were measured comparing SKOV-3 cells with reduced claudin-1 to controls. SKOV-3 cells with reduced claudin-1 were also examined without exogenous LPA to determine their ability to heal a wounded cell monolayer. We detected an inability to seal wounded monolayers within 24 h, a process requiring LPA. We also detected an abundance of detached cells floating throughout the medium in wells with reduced claudin-1 expression (Figure 5C). It is unclear what percentage of suspended cells observed in the gap between monolayers were attached (reseeded cells) or not (floating cells unable to reattach through loss of claudin-1), but most cells were rounded and did not exhibit flattened morphology associated with attachment. Reducing claudin-1 expression resulted in a 9-fold and 10-fold increase in the number of suspended cells detected in the space between monolayers compared to Mock and RISC-free siRNA control conditions, respectively (Figure 5D). These responses to claudin-1 siRNA could not be attributed to variation of viability alone (Figure 5E) and suggests that reducing claudin-1 allows SKOV-3 cells to detach from confluent layers and float in suspension.

We compared the migratory capabilities of SKOV-3 cells with Mock, RISC-free and claudin-1 siRNA-transfection. In response to LPA (20 µM) stimulation in a Boyden chamber, an inhibition in LPA-stimulated migration was observed in cells with reduced claudin-1 compared to both Mock and RISC-free siRNA control transfected cells (Figure 5F and G). The data suggests claudin-1 enhances LPA-mediated Boyden chamber migration in SKOV-3 cells and may reflect an inability of cells to attach to the substratum required for Boyden chamber migration through pores. This is a significant effect because motility is a major effect of LPA on SKOV-3 cells.

## Discussion

Herein we present several important findings: first, a 39-gene, LPA-mediated signature identifies serous EOC as being different from with other types of EOC; second, the 39-gene signature correlates with worsened prognosis; third, a group of 7 genes is sufficient to categorize the LPA signature; fourth, a single biomarker, claudin-1, is increased in serous EOC and claudin-1 mRNA levels are increased by LPA; fifth, LPA induced claudin-1 localization to the plasma membrane; sixth, claudin-1 is involved in LPA-mediated adhesion and migration. The 39-gene expression signature was defined using OVCAR-3, an ascites-derived serous EOC cell line. Given that elevated levels of LPA are present in the vast majority of ascites fluids, OVCAR-3 is likely to have been exposed to LPA in the tumor microenvironment, making it an ideal model. It is intriguing that LPA stimulation was necessary in order to define a gene expression profile reflective of serous ovarian cancers suggesting that in vivo serous ovarian cancers are exposed and responding to LPA. A number of components in the 39-gene signature have been identified in previous ovarian candidate gene studies [39]–[41] and other genes have known associations with LPA [30]–[35]. However, the signature contains transcripts previously unassociated with LPA, such as *CLDN1*, and additional experiments are underway to elucidate these connections.

Using gene expression analysis and mining through ovarian cancer patient datasets, we demonstrate that the 39-gene signature characterizes serous EOC and a worsened prognosis. Comparing disease specific survival and progression free survival of LPA-signature-positive versus dissimilar groups, the LPA-positive patients exhibit a significantly reduced time of survival. This is remarkable considering that the preeminent biomarker among the 39-gene signature, claudin-1, was unfortunately absent from this ovarian dataset.

Additionally, the worsened prognosis classified by the 39-gene signature is observed when the comparison is made between serous samples in different disease stages. Among serous specimens, the LPA-positive group had a greater percentage of stage IIIc serous carcinoma than stage IIIa and IIIb. This observation is indicative of prognosis because the 5-year survival for stage IIIa and IIIc carcinomas is 41.4% and 23.4%, respectively.

The fact that claudin-1 was not included in every prognosis dataset we analyzed indicates that the prognostic ability of the 39-gene signature is not reliant upon on any singular gene expression change, but rather likely represents the conglomerate result of systematic changes resulting from LPA-induced changes in RNA levels. We did, however, observe an ability to recapitulate the serous EOC clustering using an abbreviated 7-gene signature that included *CLDN1*, *CYR61*, *FN1*, *IL-8*, *MUC1*, *TNC* and *THBS1*. Using these seven drivers, similar (and even enhanced) results were achieved compared to using all 39-genes. It is possible that these seven along with a few others (i.e. *ATF3*, *EGR1*, *CXCL1*, etc.) represent the majority of the effects mediated by LPA in the tumor microenvironment and that the others represent non-specific, growth-factor induced transcriptional activity that could be related to homeostatic regulation. Among signature drivers and the seven drivers changes, many have known associations with LPA, including *CXCL1* [30], *CYR61* [31], *EGR1* [32], *IL-8* [33], *FN1* [34] and *PLAU* [35].

In this study *CLDN1* is elevated in serous EOC and sufficient to cluster ovarian tumor types, which suggests it, by itself, is an ovarian cancer-type biomarker but not a prognostic biomarker. This is in agreement with a previous study that distinguished between endometrioid and serous carcinoma using immunohistochemistry and claudin 1–7 expression [42]. Another independent study found claudin-1, claudin-3 and claudin-7 up-regulation together in ovarian cancer effusions predicts poor progression-free and overall survival [43]. Unlike the aforementioned studies, ours deviates because we use gene expression profiling, claudin-1, but not claudin-3, -4 or -7 and we also include normal, mucinous and clear cell tumors to further suggest that claudin-1 could distinguish serous from the other histologic types. We were unable to assess a prognostic ability of claudin-1 alone, in part because claudin-1 was not available on the earlier microarray profiling used to collect some of the patient data for analyses.

This study reports the novel observations that LPA markedly increases claudin-1 transcription and enhances its localization to the plasma membrane, likely into tight junctions. Cells lose adhesion with the loss of tight junctional proteins, like claudins, thus allowing the acquisition of migratory capabilities [44]. With reduced claudin-1 expression, the data shows a decrease in OVCAR-3 adhesion, an increase in the potential of SKOV-3 cells to detach and float in medium and a decrease in LPA-mediated migration of SKOV-3 through a Boyden chamber. The latter is likely the result in an inability of siClaudin-1 cells to adhere to the Boyden chamber substratum required to migrate through the pores.

If we rationalize the data acquired in this study, what broader influence does the LPA-mediated increase in claudin-1 have during the progression of ovarian cancer? The answer may arise out of the kind of disease spread that occurs—ovarian cancer spreads intraperitoneally. Many cancers that progress to metastatic tumors must acquire the ability to degrade the extracellular matrix, adhere to other cells, perform intravasion, survive turbulent anoikis conditions in the circulation and complete extravasion to invade new tissues. The ovaries are located in the peritoneal cavity, creating a uniquely passive, and comparatively uncomplicated, metastasis scenario. Malignant ovarian tumor cells floating in ascites fluid or peritoneal washings are detected in stage Ic ovarian cancer and above [45], making cells shed from the primary ovarian tumor commonly observed in this disease. These shed cells are then passively transported by peritoneal fluid and can seed on surrounding tissues if they acquire the ability to implant on peritoneal surfaces [15], [45]. What molecular proteins would be necessary to assist implantation of free-floating cells onto peritoneal surfaces? Cell adhesion molecules. Corroborating observations include the fact that normal ovarian surface epithelium rarely expresses E-cadherin [19] but ovarian carcinomas express high levels of E-cadherin and other cell adhesion molecules [46]. Further, the 39-gene signature that is rich with key cell adhesion transcripts such as: *CLDN1*, *FN1*, *MUC1*, *THBS1* and *TNC*, along with cytokines that promote or regulate adhesion, *CYR61* and *IL-8*.

In summary, we have identified an LPA-driven, 39-gene signature that is sufficient on unsupervised clustering to predict worsened outcome in ovarian cancer. While some of the effect on outcome could be related to the signature enriched in serous epithelial tumors that are associated with a worsened outcome, this appears insufficient to explain the overall effect on tumor behavior. Thus, an LPA signature likely reflective of LPA function in ovarian cancer patients is highly enriched in a population of ovarian cancers. This could prove to be important in determining prognosis, in response to current therapy or in selecting patients for therapy targeting LPA. Claudin-1 is a leading candidate biomarker for such selection as LPA-targeted therapies move forward to clinical trials. This is particularly important as a series of inhibitors targeting LPA signaling, LPA production and LPA receptors are indeed in preclinical development [36]. In addition, the demonstration herein that LPA signaling alters outcome in ovarian cancer increases the impetus for clinical trials of drugs targeting this pathway.

## Supporting Information

Figure S1 Comparison and validation of the LPA-induced gene expression transcriptional profile in OVCAR-3 cells. Pairwise scatter plots demonstrate the microarray gene expression values of the triplicate samples under one experimental condition of either (A) control or (B) LPA-stimulated (18∶1, 20 µM for 24 h) or (C) EGF-stimulated (20 µM for 24 h). In the first row of three plots the line represents sample 1 with itself (hyb.1), sample 1 with sample 2 (hyb.2) and sample 1 with sample 3 (hyb.3). The comparisons continue with the other rows so that the three plots on the upper right half are mirrors of the three in the lower half. Because the corresponding dots fall close within a 45° reference line, the replicated microarray chips are highly correlated, suggesting the reproducibility of each condition is high. (D) Quantitative RT-PCR of OVCAR-3 cells stimulated without or with LPA (20 µM) for the times indicated. The mRNA was extracted from OVCAR-3 cells and processed to corroborate transcript changes seen on the microarray. The data shown is the average of results from transcription factors and a co-repressor in OVCAR-3 and SKOV-3 cells relative to Beta-2 microglobulin. The Expression Analysis Systematic Explorer (EASE) software program was used to categorize microarray results into (E) cellular localization or (F) molecular function. The majority of genes are localized to regions affecting extracellular signaling including the plasma membrane (30%), extracellular matrix (12%) or secretion into the extracellular space (20%). A variety of molecular functions are represented in the signature with cell-cell communication (21%) and signal transduction (14%) the most prominent categories.
(1.60 MB TIF)

Figure S2 Various ovarian cancer datasets demonstrate that the 39-gene signature characterizes serous EOC. (A) The patient dataset GSE6822 (N = 74) [25] was examined with the genes available contained in the 39-gene signature and hierarchical clustering separated a group (N = 19) that was strongly positive for the LPA-signature (far right cluster) and corresponded most closely with invasive serous EOC. (B) The patient dataset GSE10971 (N = 37) was divided into two groups based upon similarity to the LPA signature as determined by increased expression and hierarchical clustering (data not shown). All patients in the LPA-positive cluster (100%) had high-grade serous carcinoma while the majority of patients in the dissimilar cluster (N = 24 out of 25) had non-malignant fallopian tube epithelium carcinoma.
(1.79 MB TIF)

Figure S3 CLDN-1 and cell adhesion-related proteins drive the clustering of the LPA transcriptomic signature. (A) The patient dataset, GSE6008, N = 103, was downloaded from the NCBI Entrez GEO DataSets website and analyzed using a previously-identified set of drivers, CLDN1, CYR61, FN1, IL-8, MUC1, THBS1, and TNC. These genes were capable of classifying the LPA-positive serous cluster of patient samples without the other genes comprising the 39-gene signature. Specimens (N = 79) isolated from patients treated at the Norwegian Radium Hospital re-examined by hierarchical clustering using only those available genes with a role in cell adhesion. Kaplan-Meier analysis of the different clusters indicates that the LPA-signature positive cluster has shorter median values for both disease-specific (B) and progression-free (C) survival compared to the LPA-negative cluster. (D) Out of the seven drivers in A, claudin-1 (CLDN1) was sufficient to differentiate serous from other EOC types. Serous EOC highly expresses CLDN1 whereas mucinous tumors, clear cell tumors and normal specimens were low and the majority of endometrioid samples are at the median level or below (N = 32, 89%).
(2.23 MB TIF)
