# Supplementary figures and images for: Lysophosphatidic Acid-Induced Transcriptional Profile Represents Serous Epithelial Ovarian Carcinoma and Worsened Prognosis
Source: PLoS One. 2009 May 15;4(5):e5583. doi: 10.1371/journal.pone.0005583 (PMC2679144; doi:10.1371/journal.pone.0005583)

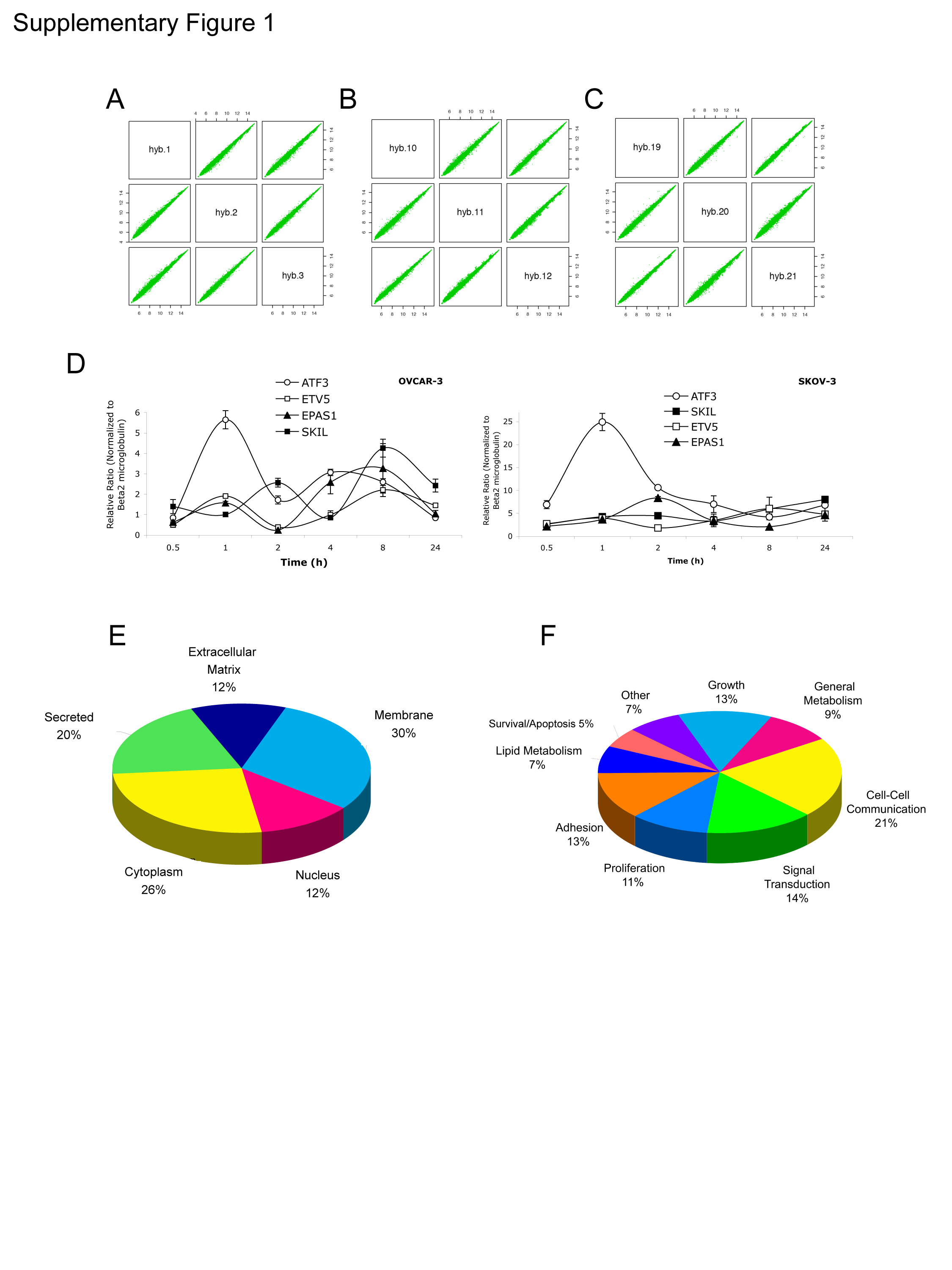

Supplement: Figure S1 — Comparison and validation of the LPA-induced gene expression transcriptional profile in OVCAR-3 cells. Pairwise scatter plots demonstrate the microarray gene expression values of the triplicate samples under one experimental condition of either (A) control or (B) LPA-stimulated (18∶1, 20 µM for 24 h) or (C) EGF-stimulated (20 µM for 24 h). In the first row of three plots the line represents sample 1 with itself (hyb.1), sample 1 with sample 2 (hyb.2) and sample 1 with sample 3 (hyb.3). The comparisons continue with the other rows so that the three plots on the upper right half are mirrors of the three in the lower half. Because the corresponding dots fall close within a 45° reference line, the replicated microarray chips are highly correlated, suggesting the reproducibility of each condition is high. (D) Quantitative RT-PCR of OVCAR-3 cells stimulated without or with LPA (20 µM) for the times indicated. The mRNA was extracted from OVCAR-3 cells and processed to corroborate transcript changes seen on the microarray. The data shown is the average of results from transcription factors and a co-repressor in OVCAR-3 and SKOV-3 cells relative to Beta-2 microglobulin. The Expression Analysis Systematic Explorer (EASE) software program was used to categorize microarray results into (E) cellular localization or (F) molecular function. The majority of genes are localized to regions affecting extracellular signaling including the plasma membrane (30%), extracellular matrix (12%) or secretion into the extracellular space (20%). A variety of molecular functions are represented in the signature with cell-cell communication (21%) and signal transduction (14%) the most prominent categories. (1.60 MB TIF) [file pone.0005583.s001.tif]

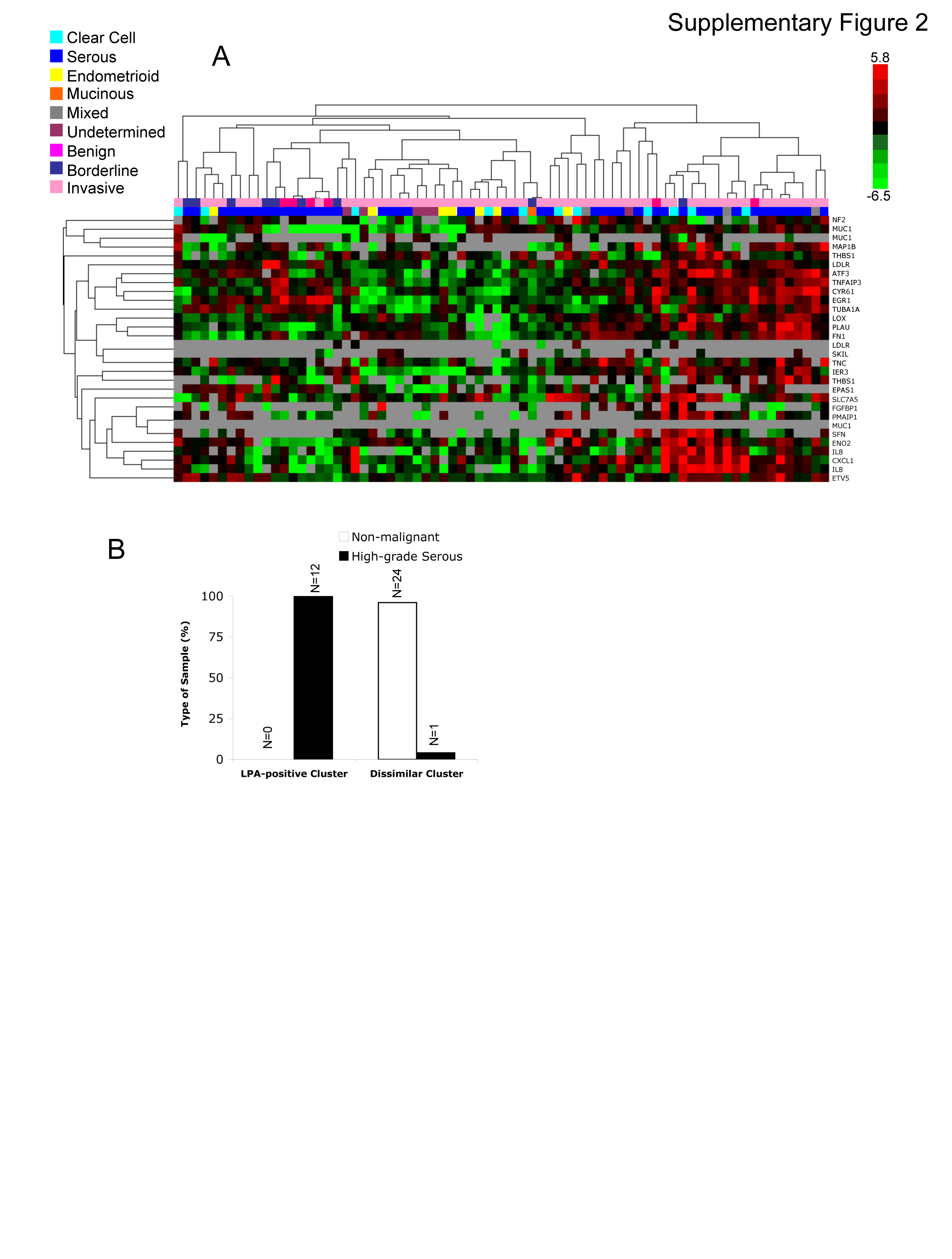

Supplement: Figure S2 — Various ovarian cancer datasets demonstrate that the 39-gene signature characterizes serous EOC. (A) The patient dataset GSE6822 (N = 74) [25] was examined with the genes available contained in the 39-gene signature and hierarchical clustering separated a group (N = 19) that was strongly positive for the LPA-signature (far right cluster) and corresponded most closely with invasive serous EOC. (B) The patient dataset GSE10971 (N = 37) was divided into two groups based upon similarity to the LPA signature as determined by increased expression and hierarchical clustering (data not shown). All patients in the LPA-positive cluster (100%) had high-grade serous carcinoma while the majority of patients in the dissimilar cluster (N = 24 out of 25) had non-malignant fallopian tube epithelium carcinoma. (1.79 MB TIF) [file pone.0005583.s002.tif]

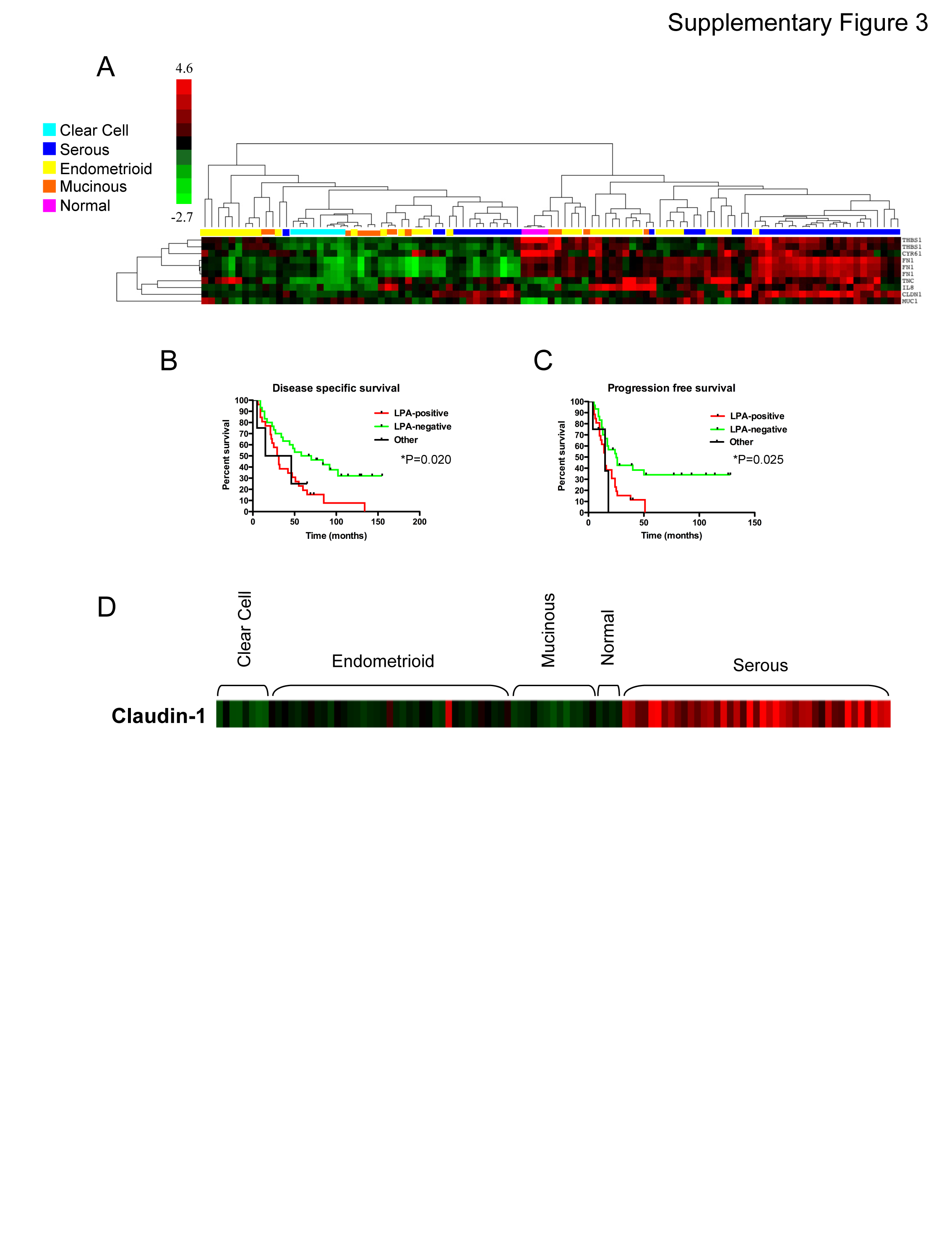

Supplement: Figure S3 — CLDN-1 and cell adhesion-related proteins drive the clustering of the LPA transcriptomic signature. (A) The patient dataset, GSE6008, N = 103, was downloaded from the NCBI Entrez GEO DataSets website and analyzed using a previously-identified set of drivers, CLDN1, CYR61, FN1, IL-8, MUC1, THBS1, and TNC. These genes were capable of classifying the LPA-positive serous cluster of patient samples without the other genes comprising the 39-gene signature. Specimens (N = 79) isolated from patients treated at the Norwegian Radium Hospital re-examined by hierarchical clustering using only those available genes with a role in cell adhesion. Kaplan-Meier analysis of the different clusters indicates that the LPA-signature positive cluster has shorter median values for both disease-specific (B) and progression-free (C) survival compared to the LPA-negative cluster. (D) Out of the seven drivers in A, claudin-1 (CLDN1) was sufficient to differentiate serous from other EOC types. Serous EOC highly expresses CLDN1 whereas mucinous tumors, clear cell tumors and normal specimens were low and the majority of endometrioid samples are at the median level or below (N = 32, 89%). (2.23 MB TIF) [file pone.0005583.s003.tif]
